# Short versus standard duration antibiotic therapy for acute streptococcal pharyngitis in children

**DOI:** 10.1590/S1516-31802010000100013

**Published:** 2010-01-07

## Abstract

**BACKGROUND:**

The standard duration of treatment for acute group A beta hemolytic streptococcus (GABHS) pharyngitis with oral penicillin is 10 days. Shorter duration antibiotics may have comparable efficacy.

**OBJECTIVES:**

To summarize the evidence regarding the efficacy of two to six days of newer oral antibiotics (short duration) compared to 10 days of oral penicillin (standard duration) in treating children with acute GABHS pharyngitis.

**SEARCH STRATEGY:**

We searched the Cochrane Central Register of Controlled Trials (CENTRAL) (The Cochrane Library 2007, issue 4), which contains the Acute Respiratory Infections Group’s Specialized Register; the Database of Abstracts of Reviews of Effects (DARE); MEDLINE (1966 to October 2007); OLDMEDLINE (1950 to December 1965); and EMBASE (January 1990 to November 2007).

**SELECTION CRITERIA:**

Randomized controlled trials (RCTs) comparing short duration oral antibiotics to standard duration oral penicillin in children aged 1 to 18 years with acute GABHS pharyngitis.

**DATA COLLECTION AND ANALYSIS:**

Two review authors scanned the titles and abstracts of retrieved citations and applied the inclusion criteria. We retrieved included studies in full and extracted data. Two review authors independently assessed trial quality.

**MAIN RESULTS:**

Twenty studies were included with 13,102 cases of acute GABHS pharyngitis. Compared to standard duration treatment, the short duration treatment had shorter periods of fever (mean difference (MD) -0.30 days, 95% CI -0.45 to -0.14) and throat soreness (MD -0.50 days, 95% CI -0.78 to -0.22); lower risk of early clinical treatment failure (OR 0.80, 95% CI 0.67 to 0.94); no significant difference in early bacteriological treatment failure (OR 1.08, 95% CI 0.97 to 1.20), or late clinical recurrence (OR 0.95, 95% CI 0.83 to 1.08). However, the overall risk of late bacteriological recurrence was worse in the short duration treatment (OR 1.31, 95% CI 1.16 to 1.48), although no significant differences were found when studies of low dose azithromycin (10mg/kg) were eliminated (OR 1.06, 95% CI 0.92 to 1.22). Three studies reported long duration complications with no statistically significant difference (OR 0.53, 95% CI 0.17 to 1.64).

**AUTHORS’ CONCLUSIONS:**

Three to six days of oral antibiotics had comparable efficacy compared to the standard duration 10 day oral penicillin in treating children with acute GABHS pharyngitis. In countries with low rates of rheumatic fever, it appears safe and efficacious to treat children with acute GABHS pharyngitis with short duration antibiotics. In areas where the prevalence of rheumatic heart disease is still high, our results must be interpreted with caution.

Altamimi S, Khalil A, Khalaiwi KA, Milner R, Pusic MV, Al Othman MA. Short versus standard duration antibiotic therapy for acute streptococcal pharyngitis in children. Cochrane Database of Systematic Reviews 2009, Issue 1. Art. No.: CD004872. DOI: 10.1002/14651858.CD004872.pub2.

FURTHER INFORMATION: Centro Cochrane do Brasil Rua Pedro de Toledo, 598 Vila Clementino — São Paulo (SP) — Brasil CEP 04039-001 Tel. (+55 11) 5579-0469/5575-2970 http://www.centrocohranedobrasil.org.br/

This section was edited under the responsibility of Brazilian Cochrane Centre

The full review is available (free access) from http://cochrane.bvsalud.org/portal/php/index.php?lang=pt.

